# Acetyl-L-Carnitine as an Add-On Treatment in Fibromyalgia Syndrome: A Retrospective Analysis on 183 Patients, According to the Generalized Linear Mixed Model for Longitudinal Data

**DOI:** 10.3390/biomedicines13040820

**Published:** 2025-03-28

**Authors:** Vittorio Schweiger, Libera Villagrossi, Francesco Taus, Leonardo Gottin, Eleonora Bonora, Marco Anderloni, Giustino Varrassi, Luca Polati, Marta Nizzero, Alvise Martini, Enrico Polati

**Affiliations:** 1 Anesthesiology, Intensive Care and Pain Therapy Center, Department of Surgery, Dentistry and Maternal Sciences, University of Verona, 37134 Verona, Italy; libera.villagrossi@gmail.com (L.V.); leonardo.gottin@univr.it (L.G.); eleonora.bonora@aovr.veneto.it (E.B.); marco.anderloni@studenti.univr.it (M.A.); pola.luca@hotmail.it (L.P.); marta.nizzero@gmail.com (M.N.); alvise.martini@univr.it (A.M.); enrico.polati@univr.it (E.P.); 2 Department of Diagnostics and Public Health, Section of Epidemiology and Medical Statistics, University of Verona, 37134 Verona, Italy; francesco.taus@univr.it; 3 Paolo Procacci Foundation, 00193 Rome, Italy; giuvarr@gmail.com

**Keywords:** fibromyalgia, Acetyl-L-carnitine, pain, quality of life

## Abstract

**Background**. Fibromyalgia Syndrome (FMS) is characterized by chronic widespread pain, sleep disturbances, fatigue and cognitive impairment. **Methods**. In this retrospective study, we analyzed data collected between 2017 and 2022 regarding Acetyl-L-Carnitine (ALC) as an add-on treatment in 183 adult patients with FMS according to the 2016 ACR (American College of Rheumatology) criteria and patients’ pain lasting for over three months. Patients with prior exposure to ALC or without informed consent were excluded. **Results**. Regarding efficacy, in the 137 analyzed patients, the change from baseline to the end of observation in Visual Analogue Scale score (VAS) was statistically significant, ranging from 75.9 ± 1.56 to 51.9 ± 1.99 (*p* < 0.001). Patients without FMS concomitant drug treatments achieved better VAS reduction than patients who were not drug-free at baseline. Regarding quality of life, a significant improvement in the Revised Fibromyalgia Impact Questionnaire (FIQ-R) score was evidenced, ranging from 75.1 ± 1.13 to 53.5 ± 1.34 (*p* < 0.001). The Short Form 12 Health Survey (SF12) scores showed a statistically significant improvement in both physical and mental components. Finally, the Pittsburgh Sleep Quality Index (PSQI) did not show a statistically significant difference from baseline. In the whole population, 23 patients (16.7%) reported Adverse Events (AEs), predominantly insomnia, shivering, headaches, and nausea. Only six patients reporting AEs discontinued the ALC treatment. **Conclusions**. This retrospective study evidenced the efficacy and safety of ALC in FMS patients. This may represent a useful approach, particularly for long-term treatments. Methodologically stronger studies will be necessary to validate our observations.

## 1. Introduction

Fibromyalgia syndrome (FMS) is a condition characterized by chronic widespread pain, along with sleep disturbances, fatigue and cognitive impairment [1]. The prevalence of FMS is estimated to range between 2% and 8% of the adult population worldwide, with a strong preference for the female gender. To date, FMS is considered the leading cause of widespread pain in females aged 20 to 50 years [2,3]. FMS belongs to a group of overlapping conditions defined as “central sensitivity syndromes” that include Chronic Fatigue Syndrome, Irritable Bowel Syndrome, Bladder Pain Syndrome, Temporo-mandibular Joint Disorder, Tension-Type Headache and others [4]. Currently, FMS Treatment Guidelines highlight the importance of a multimodal approach, starting from non-pharmacological therapies, and adding pharmacological approaches only for non-responsive patients [5]. Among these treatments, in patients with severe pain, duloxetine, pregabalin or tramadol (alone or in combination with paracetamol) are recommended, while in patients with severe sleep problems, low doses of amitriptyline, cyclobenzaprine or pregabalin at night were suggested [5]. Nevertheless, the vitamin D deficiency highlighted in some FMS patients may represent an interesting target for vitamin D supplementation for pain and QoL improvement, as demonstrated in a recent study [6]. However, current evidence does not demonstrate significant benefits from drug treatments alone or in combination in FMS, with only a minority of patients experiencing clinical improvement or a high rate of Adverse Events (AEs) and dropouts [7]. From a pathophysiological perspective, FMS is regarded as a model of nociplastic pain, where pain arises from an imbalance between ascending and descending pathways. Other mechanisms, including immune, endocrine, genetic and psychosocial factors, have also been considered [8,9]. Additionally, neuroinflammation and oxidative stress imbalance play a significant role in FMS pathophysiology [10,11]. In addition, great relevance in FMS maintenance has also been attributed to psychological disturbances [12]. While several antidepressant drug therapies have been evaluated in FMS treatment, also with novel modalities of delivery at the CNS level [13], experimental studies have highlighted a potential benefit of Acetyl-L-carnitine (ALC), not only as a pain-relieving drug but also as modulator of neuroinflammation, oxidative stress and psychological status [14]. ALC is the acetylated derivative of the amino acid L-carnitine and plays a pivotal role in cellular energy production, facilitating the beta-oxidation process of long-chain fatty acids within the mitochondria [14]. ALC has interesting properties at the CNS level, like neuroprotection and an anti-depressant effect [15,16]. ALC may also have a role in mitigating oxidative stress in several tissues [17]. Moreover, recent evidence on the association between FMS severity and carnitine deficiency, which lead to muscular weakness, fatigue and intolerance to physical exercise, may justify ALC treatment in these patients, to improve physical performance and muscle function [17]. The aim of this retrospective study was to evaluate the efficacy and safety of ALC as an add-on treatment in a population of FMS patients referred to a Pain Centre in Italy.

## 2. Materials and Methods

### 2.1. Study Design

In this retrospective study, we analyzed the data routinely collected during daily clinical practice in our Pain Therapy Centre, regarding patients aged ≥ 18 yrs. with FMS diagnosis according to the ACR (American College of Rheumatology) revised criteria 2016 (symptoms for at least 3 months, WPI ≥ 7 and SS ≥ 5 or WPI 4–6 and SS ≥ 9) [18] and pain > 40 according to the VAS score (Visual Analogue Scale 0–100), who, between 2017 and 2022, were prescribed ALC, regardless of the concomitant pharmacological FMS therapy (add-on treatment). The ALC (Nicetile^®^, Alfasigma S.p.A, Bologna, Italy) was prescribed starting from intramuscular injection, with 500 mg ampoules once daily for 10 days, followed by oral administration of 500 mg tablets twice daily for 20 days, and then a maintenance treatment of a 500 mg tablet once daily for 14 months. Exclusion criteria were a known allergy to the drug or its excipients, previous exposure to ALC or lack of informed consent for treatment.

### 2.2. Efficacy and Safety Assessments

The primary efficacy endpoint was the change in Visual Analogue Scale (VAS) scores from baseline to the end of the study period. The VAS, a numerical rating scale ranging from 0 to 100, employs a straight line without interruptions, with endpoints delineating extremes from no pain (0) to the worst imaginable pain (100). The patient marks the point corresponding to is/her pain level on the line, which is then measured by the clinician using a millimetre scale. The VAS scale is easily administered by the doctor who performs the examination, is well-received by patients, and is commonly adopted in our clinical practice as the primary measure for assessing pain treatment outcomes [19]. The secondary efficacy endpoints were the change in the Fibromyalgia Impact Questionnaire—Revised (FIQ-R, Italian version) score, in the Short Form 12 (SF12) score and in the Pittsburgh Sleep Quality Index (PSQI) score from baseline to the end of the observation period. The FIQ-R proves highly valuable for assessing quality of life (QoL) in FMS patients, displaying notable sensitivity in clinical studies and evaluating several aspects in FMS, such as physical functioning, work status, depression, anxiety, sleep, pain, stiffness, fatigue, and well-being [20] (the supplementary data can be accessed through the link provided in the Supplementary Materials). FIQ-R values range from 0 to 100. Higher scores indicate worse QoL. SF12 is derived from SF36, and can be used to explore summary measures of health status. The physical component summary (PCS) includes scales assessing physical functioning, role functioning difficulties caused by physical problems, bodily pain and general health. The mental component summary (MCS) includes scales assessing vitality, social functioning, role functioning difficulties caused by emotional problems and mental health. Higher scores represent better functioning [21]. The PSQI is a self-administered questionnaire that assesses sleep quality and disturbances over a one-month interval. Nineteen individual items generate 7 component scores such subjective sleep quality, sleep latency, sleep duration, habitual sleep efficiency, sleep disturbances, use of sleep medication, and daytime dysfunction. The 7 component scores are then summed to yield a global PSQI score, ranging from 0 to 21. Higher scores indicate worse sleep quality, and a global PSQI score ≥5 is consistent with poor sleep quality [22]. All the evaluations for assessment were administered in the form of scales or questionnaires completed by the patients without any interference by the clinicians, at baseline and during the subsequent visits, as routine evaluations. Safety assessments encompassed monitoring the frequency of treatment-related Adverse Events (AEs), while also documenting other reasons leading to the discontinuation of therapy.

### 2.3. Ethics

All the study procedures adhered to the Helsinki Declaration (1975/83). The study was approved by the local Ethical Committee (RED Register for Retrospective Studies, 1751CESC).

### 2.4. Data Collection and Statistical Analysis

We collected all relevant information about patients undergoing ALC treatment from clinical records associated with scheduled institutional medical examinations for chronic pain patients. Demographic, medical and clinical characteristics were summarized using descriptive statistics and represented in table form (see Table 1). Our analysis focused on FMS patients undergoing ALC treatment with VAS, FIQ-R, SF12 and PSQI scores documented at the baseline visit and during at least one of the subsequent medical assessments. Evaluations of VAS and FIQ-R were collected at baseline, after 1, 2 and 3 months from the start of treatment, and subsequently every 3 months throughout the 15-month maintenance therapy. Evaluations of SF12 and PSQI were collected at baseline and after 3 and 6 months from the start of treatment. All these evaluations are performed and registered routinely as a part of the usual care of FMS patients in our Centre. Primary and secondary efficacy endpoints were examined using a Generalized Linear Mixed Model (GLMM). This analysis of longitudinal data allows us to accommodate a range of response variables, including continuous, binary or count data, providing versatility in analyzing different outcomes [23,24]. The GLMM structure allowed us to appropriately account for within-subject variance, handle missing observations, and model the dependence of repeated measurements within the same patient. Covariates such as gender, age (grouped in quartiles), and concomitant therapies (Yes/No) were used to adjust the model. Post hoc analyses were conducted to compare adjacent time points (e.g., baseline vs. 1 month, 1 month vs. 3 months, etc.). Differences between time points were tested using adjusted *p*-values to account for multiple comparisons, and the Bonferroni correction was applied. A two-sided *p* < 0.05 was considered statistically significant for all analyses, and data were analyzed using STATA Statistical Software, 18 (StataCorp, College Station, TX, USA).

## 3. Results

### 3.1. Study Population

**Figure 1:**
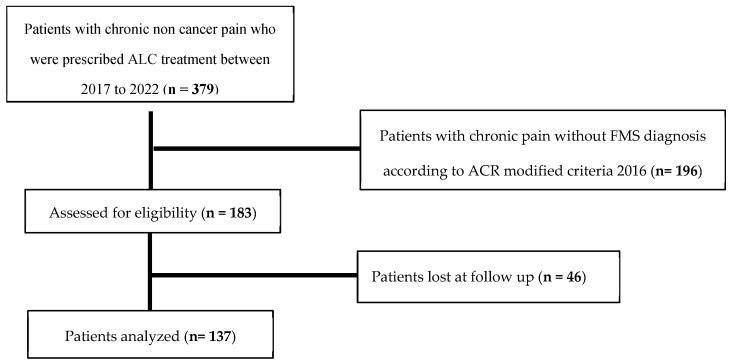
Flow diagram of the study population.

From 2017 to 2022, ALC treatment was prescribed to 379 individuals experiencing chronic non-cancer pain at our Pain Centre. Among these, 183 patients were identified as suffering from FMS based on the ACR revised criteria 2016. Of these, 46 patients (25.1%) did not return to any medical evaluation after treatment prescription, and were therefore considered lost to follow-up and excluded from the analysis. The flow diagram of the study population is represented in Figure 1.

The information about patient demographics and baseline characteristics is summarized in Table 1.

**Figure 2:**
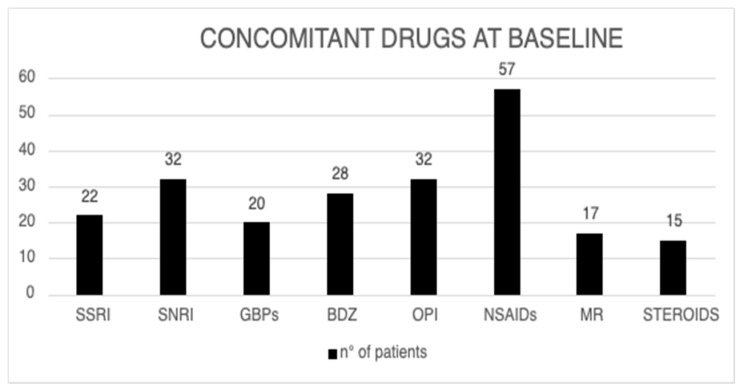
Baseline pharmacological drug treatments in the eligible population (SSRI = Selective Serotonin Reuptake Inhibitors; SNRI = Serotonin–Noradrenaline Reuptake Inhibitors; GBPs = Gabapentinoids; BZD = Benzodiazepine; OPI = Opioid; NSAIDs = Non-steroidal anti-inflammatory drugs; MR = Muscle Relaxant).

Details on concomitant FMS drugs consumption at baseline are reported in Figure 2.

### 3.2. Efficacy

**Figure 3:**
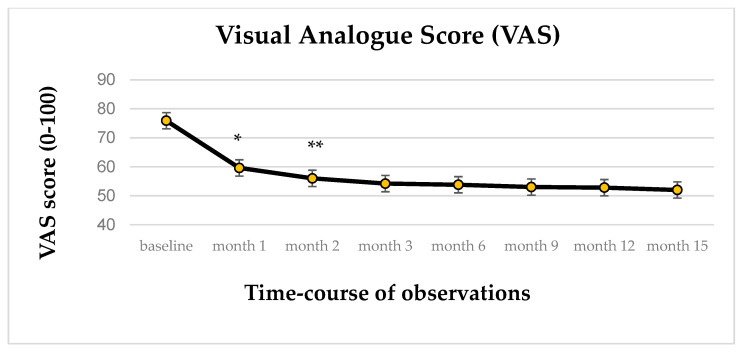
Time-course of VAS score value (IC95%); * *p* < 0.001; ** *p* < 0.05.

Regarding the primary endpoint, in the 137 analyzed patients, the change over time in VAS score (mean ± standard error, S.E.) was statistically significant (*p* < 0.001), ranging from 75.9 ± 1.56 at baseline to 51.9 ± 1.99 at study end (Figure 3). The covariates sex and concomitant therapies did not have a significant effect, while a statistically significant difference was observed in patients aged between 53 and 60 years. Notably, these patients are reported to have a lower VAS value than the reference group (20–44 years old). The evaluation for multiple comparisons, using post hoc analysis, showed a significant decrease (*p* < 0.001) in the pain intensity at the first month of treatment compared with the baseline. The VAS mean scores changed from 75.9 ± 1.56 at baseline to 59.6 ± 1.67 at first follow-up. This value persisted almost constantly during the remaining observational period.

**Figure 4:**
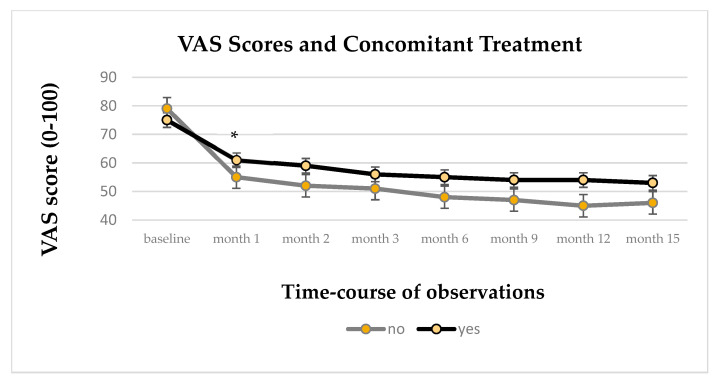
Time-course of VAS score value (IC95%) in patients with ALC treatment with or without concomitant drug therapies; * *p* < 0.001. yes = concomitant therapy; no = drug-free.

Interactions between the covariates sex; age, in quartiles; and concomitant drug treatment during the ALC treatment period were also tested. Only the variable concomitant drug showed an interaction with treatment times, specifically within the first month of treatment (*p* < 0.001). In the first month of ALC treatment, drug-free patients achieved lower VAS values than patients with drug treatments despite having slightly higher VAS values at baseline. Over time, the trend in VAS estimates remained constant between the two groups (Figure 4).

**Figure 5:**
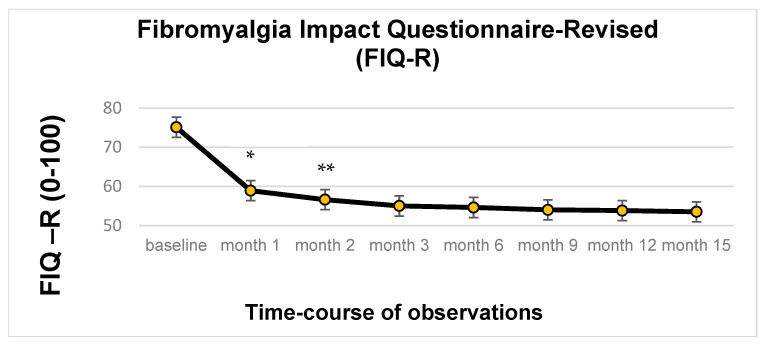
Time-course of FIQ-R score value (IC95%): * *p* < 0.001; ** *p* < 0.05.

Regarding the secondary endpoints, the analysis of 137 patients revealed a statistically significant change over time in the FIQ-R score (*p* < 0.001), ranging from 75.1 ± 1.13 at baseline to 53.5 ± 1.34 at study end. Notably, none of the covariates (gender, age or concomitant drug therapies) had a significant effect. In the post hoc analysis, a statistically significant decrease (*p* < 0.001) in the mean FIQ-R value was observed at the first follow-up compared to the baseline, changing from 75.1 ± 1.13 to 58.9 ± 1.18. The reduction in FIQ-R score continued over time, in a statistically significant way (*p* < 0.05) at the follow-up for the second month of treatment, compared with the previous one, decreasing from 58.9 ± 1.18 to 56.6 ± 1.23. No other significant differences were shown at subsequent follow-up times (Figure 5).

**Figure 6:**
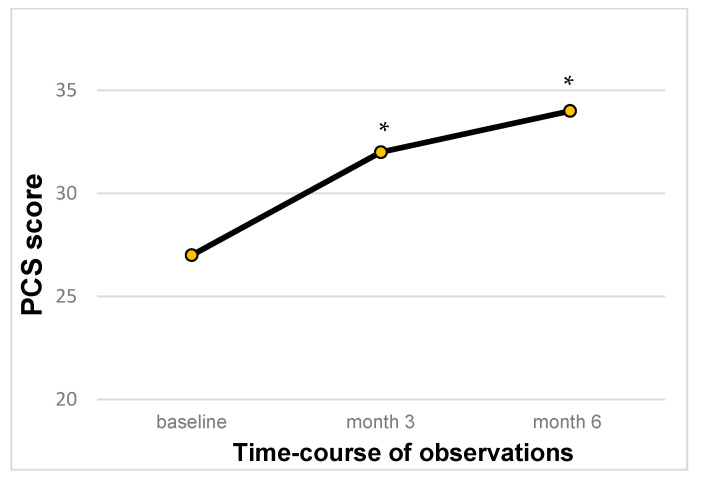
Time-course of PCS of SF12 score value (IC95%); * *p* < 0.001.

No statistically significant interactions between the covariates sex, age, and concomitant drug treatment within the duration of ALC treatment were observed. Regarding the other secondary endpoints, PSQI values analysis conducted at 3 and 6 months revealed non-statistically significant differences from baseline. On the other hand, SF12 scores showed a statistically significant difference in both physical and mental components (PCS and MCS). In particular, the PCS increased from baseline to the observations at 3 and 6 months, in a statistically significant manner, for both time points (*p* < 0.001) (Figure 6).

**Figure 7:**
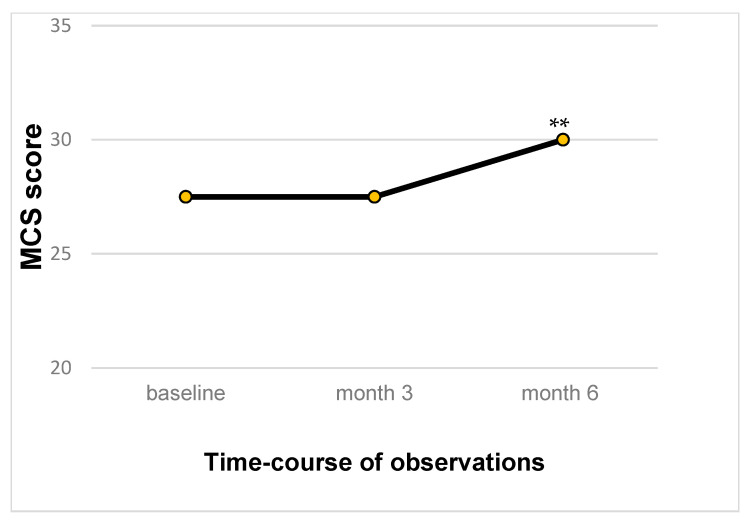
Time-course of MCS of SF12 score value (IC95%), ** *p* < 0.05.

Also, the MCS increased from the baseline, but only at the 6-month time point, in a statistically significant manner (*p* < 0.01) (Figure 7).

### 3.3. Treatment Discontinuation

Overall, 80 patients (58.4%) discontinued ALC treatment during the observation period. Notably, only 6 patients (7.5%) discontinued the treatment due to AEs, while 52 patients (65%) discontinued due to a perceived lack of efficacy, and 11 patients (8, 7%) discontinued for economic reasons (Table 2).

**Figure 8:**
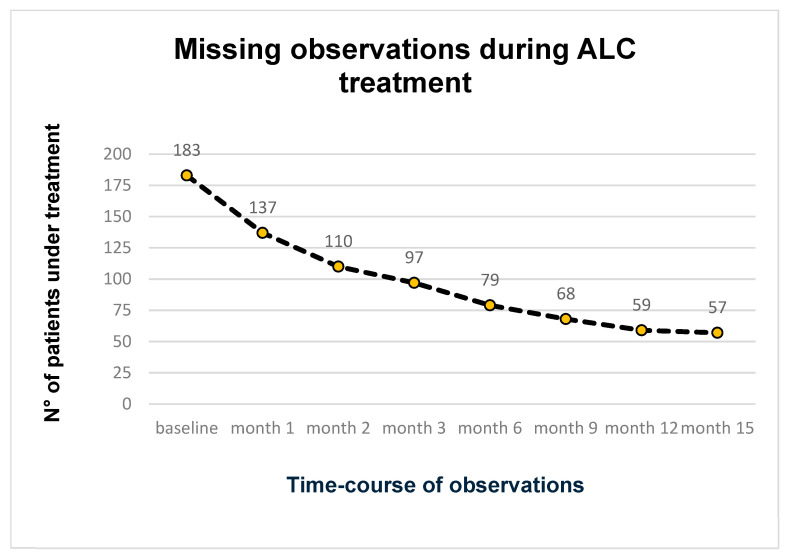
Time-course of missing observations during ALC treatment.

The course of discontinuation during observation is summarized in Figure 8.

### 3.4. Safety

In the whole population, 23 patients (16.7%) reported AEs. Regarding the type of AEs, 18 patients (13.1%) reported insomnia, 15 patients (10.9%) reported widespread shivering, 10 patients (7.3%) reported headache, and 5 patients (3.6%) reported nausea (Table 3). Only 6 patients reporting AEs discontinued the ALC treatment.

## 4. Discussion

This retrospective observational study, focusing on the use of ALC as add-on treatment in patients with FMS, revealed a long-term and statistically significant reduction in VAS scores and improvements in QoL as measured by FIQ-R. In fact, at the end of the observation period, the improvement in FIQ-R scores indicated a shift in the severity level from severe (a score between 59 and 100) to moderate (a score between 39 and 58). Additionally, ALC treatment in these patients produced an improvement in SF12 score in both components (PCS and MCS), with better PCS values evidenced already at 3 months after the start of treatment. Instead, sleep quality, measured by PSQI score, has not shown any significant modification. The incidence of AEs related to ALC treatment was notably low and led to the discontinuation of therapy only in 6 patients (4.3%). This evidence is particularly relevant in a population with a reported high sensitivity to drug treatments [25]. A statistically significant interaction between concomitant pain treatments and ALC treatment efficacy was also observed. Indeed, drug-free patients showed lower estimated VAS scores than patients who underwent concomitant pain therapy. However, this finding was not confirmed for the FIQ-R values. Growing evidence showed that ALC plays a relevant role in the treatment of chronic pain states and, particularly, in neuropathic pain and FMS [16]. The antinociceptive effect of ALC is mediated by increases in the expression of metabotropic glutamate receptor 2 (mGlu2) in the regions of CNS connected with pain transmission via an epigenetic mechanism involving the acetylation of the p65/NFkB complex [26]. Moreover, in animal models, ALC has been shown to attenuate the release of pro-inflammatory mediators (IL-1β, TNFα) from activated microglia, to modulate neuroinflammation and to reduce the concentration of oxidative stress markers, increasing the concentration of glutathione (GSH), a powerful mediator with antioxidant proprieties [27,28]. Some experimental studies on rat models also evidenced a role of ALC in improving depression states due to membrane modulation and neurotransmitter regulation [29]. Lastly, some evidence outlined the involvement of metabolic alterations and carnitine deficiency that may lead to muscular weakness, fatigue and intolerance of physical exercise, contributing to the development and maintenance of FMS [15,30]. For these reasons, the use of ALC was considered for FMS patients. While the number of randomized clinical trials (RCT) conducted in humans is limited, existing studies demonstrated noteworthy results in enhancing QoL in FMS patients following ALC treatment. In a multicenter double blind/double dummy RCT, Rossini and coll. compared administration of ALC versus a placebo in 102 FMS patients according to the 1990 ACR criteria. The treatment involved 500 mg ALC capsules, with one capsule taken twice daily, or a placebo plus one intramuscular injection of either ALC 500 mg ampoules or placebo for 2 weeks. During the following 8 weeks, the patients took three capsules daily containing either 500 mg of ALC or a placebo. Outcome measures included changing in the number of positive Tender Points (TPs), the sum of measured pain thresholds (“total myalgic score”), the SF36, the VAS score for self-perceived stiffness, fatigue, tiredness on awakening, sleep, work status, muscular–skeletal pain and the Hamilton depression scale (HADS). The “total myalgic score” and the number of positive TPs declined significantly and equally in both groups, but at the 10th week, both parameters improved only in the ALC group with a statistically significant between-group difference. A statistically significant between-group difference was observed for depression and musculo-skeletal pain [31]. In a RCT comparing the effects of 60 mg/day of duloxetine and 1500 mg/day of ALC (t.i.d.) on pain, depression, anxiety and well-being in 65 female FMS patients, Leombruni and coll. highlight, in both groups, a general clinical improvement, with positive effects on pain and depressive symptoms, but neither induced a significant improvement in anxiety. Both drugs had a positive effect on the physical component of QoL, but only duloxetine improved the psychological component [32]. In a recent study, Salaffi and coll. evaluated the efficacy of supplementing pregabalin (PGB) and duloxetine (DLX) with palmitoylethanolamide (PEA) and ALC for 24 weeks in 142 FMS patients. Twenty-four weeks after randomization, the DLX + PGB + PEA + ALC group showed additional significant improvements in all outcome measures, demonstrating the effectiveness of adding PEA + ALC therapy to DLX + PGB in FMS patients [33]. However, some notes about our study must be highlighted. Firstly, the main strength is the long study follow-up (15 months), which allows for gathering information about potential changes in patients’ QoL in response to long-term ALC treatment, usually not be easily detectable in studies with shorter observation periods. Moreover, the SF12 scores showed a statistically significant difference in both PCS and MCS components, with an early improvement in PCS (detectable after 3 months), that may be particularly useful in treating FMS-related fatigue and physical exhaustion. Finally, the use of the Generalized Linear Mixed Model (GLMM) in our study allowed for a more comprehensive data analysis, considering additional covariates and their potential interactions throughout the treatment. Our findings may suggest a synergistic effect between ALC therapy and an absence of concomitant drug treatments, potentially due to reduced pharmacological interference or enhanced metabolic responses to the therapy. This evidence is original, as few studies have investigated this specific interaction, and it warrants further investigation in future clinical research.

### Study Limitations

A significant discontinuation of ALC treatment was observed in our study in the eligible population due to a lack of efficacy. This evidence is, however, well-known and consistent with previous observations in FMS patients undergoing different types of drug treatments [34]. In a recent study, the one-year discontinuation rate among newly prescribed patients was notably high, ranging from 91% for tricyclic antidepressants (TCA) to 73.7% for selective serotonin reuptake inhibitor/serotonin–norepinephrine reuptake inhibitor (SSRI/SNRI) antidepressants. Within the same observation, over half of the patients studied had medication coverage for fewer than 20% of the days of the year, and only 9.3% exhibited a very high level of adherence [35].

## 5. Conclusions

The favourable outcomes of this retrospective study on ALC treatment in FMS represent a pivotal advancement in exploring a novel pharmacological approach centred on well-tolerated molecules, particularly beneficial for patients requiring long-term therapies. The use of Generalized Linear Mixed Models in the study has allowed for more comprehensive data analysis, considering additional covariates and their potential interactions throughout the treatment.

## Figures and Tables

**Table 1:** Baseline characteristics of eligible population.

Assessed for Eligibility	183
Gender, n (%)	
Female	170 (92.9)
Male	13 (7.1)
F:M ratio	13:1
Age, yr., mean (SD)	51.3 (±11.9)
VAS, 0–100, mean (SD)	76.1 (±14.9)
FIQ-R, 0–100, mean (SD)	73.4 (±12.7)
SF 12	
PCS, 0–100, mean (SD)	29.5 (±7.3)
MCS, 0–100, mean (SD)	26.9 (±8.04)
PSQI, 0–21, mean (SD)	10.5 (±2.6)
FMS Drug free, n (%)	
Yes	51 (27.8)
No	132 (72.1)

FIQ-R = Fibromyalgia Impact Questionnaire—Revised; VAS = Visual Analogue Scale; SF12 = Short Form 12; PSQI = Pittsburgh Sleep Quality Index.

**Table 2:** Incidence and reasons for ALC treatment discontinuation in eligible population.

Treatment discontinuation, n (%)	80 (58.4%)
Lack of efficacy	52 (65%)
Economic reasons	11 (8.7%)
Unknown	11 (8.7%)
Adverse Events (AEs)	6 (7.5%)

AEs = Adverse Events related to treatment.

**Table 3:** Incidence and type of AEs during ALC treatment in the whole population (137 patients).

AEs, n (%)	23 (16.7%)
Insomnia	18 (13.1%)
Shivering	15 (10.9%)
Headache	10 (7.3%)
Nausea	5 (3.6%)

AEs = Adverse Events related to treatment.

## Data Availability

The datasets presented in this article are not readily available due to privacy restrictions.

## Supplementary Materials

The FIQ-R Questionnaire can be downloaded at https://arthritis-research.biomedcentral.com/articles/10.1186/ar2783 (accessed on 25 March 2025).

## Abbreviations

ACR	American College of Rheumatology
AEs	Adverse Events
ALC	Acetyl-L-carnitine
DLX	Duloxetine
FIQ-R	Fibromyalgia Impact Questionnaire—Revised
FMS	Fibromyalgia syndrome
GBPs	Gabapentinoids
GLMM	Generalized Linear Mixed Models
GSH	Glutathione
HADS	Hospital Anxiety and Depression Scale
MCS	Mental component summary
MR	Muscle relaxants
NSAIDs	Non-steroidal anti-inflammatory drugs
OPI	Opioids
PCS	Physical component summary
PEA	Palmitoylethanolamide
PGB	Pregabalin
PSQI	Pittsburgh Sleep Quality Index
QoL	Quality of life
SF12	Short Form 12
SNRI	Serotonin–norepinephrine reuptake inhibitor
SSRI	Selective serotonin reuptake inhibitor
TCA	Tricyclic antidepressants
VAS	Visual Analogue Scale
WPI	Widespread Pain Index
SS	Symptom Severity score
